# The London Infirmary for Epilepsy and Paralysis

**Published:** 1867-07

**Authors:** 


*The London Infirmary for Epilepsy and Paralysis.* Cases of Epilepsy with Complications; Remarks upon Treatment. Under the care of Dr. Althaus.

There are certain complications of epilepsy which have hitherto escaped the attention of pathologists, although they appear to be by no means rare, and have an influence upon the progress of the cases in which they may happen to be present, inasmuch as their neglect may defeat our other therapeutical efforts, however persevereingly followed. Amongst these complications, Dr. Althaus, in some remarks about the etiology and progress of epilepsy, mentioned chiefly the occurrence of *hay fever* and of congenital *phimosis* in males.

Hay fever has a form of catarrh in which the symptoms of spasm predominated over that of inflammation, and which was mainly due to irritation of the sentient nerves of the respiratory tract, consequent upon the inhalation of the gases arising from fresh grass and hay. The nerves suffered more in this disorder than the mucous membrane, for there were often fits of sneezing which lasted for an hour or more with scarcely any interruption, and such urgent dyspnœa as could not be explained merely by the slight catarrhal affection of the air-passages. Hay fever was in nowise a dangerous disorder, but tended considerably to irritate and lower the tone of the nervous centres; and if it appeared in an epileptic patient, it almost always meant mischief, aggravating the attacks if such still occurred, and predisposing to relapses where the attacks had been put down. Being an essentially spasmodic disorder, it was best treated by sedatives, whereby it was generally removed in a very short time. After the symptoms of irritation had subsided, the administration of tonics was usually found necessary.

Sarah S——, aged twenty-eight, single, came under treatment as an out-patient on March 21st, 1866. She has for the last five years suffered from epileptic fits, with prolonged loss of conciousness and severe convulsions. The origin of the fits she attributed to a severe attack of small-pox, which she had in the spring of 1861, and which prostrated her a great deal. She has at present from four to six epileptic fits during the week, some in the day time, and some at night. Those which occur in the day are ushered in by visions of large black specks floating about in the air; a sort of faintness then comes over her, which rapidly passes into unconsciousness and convulsions. When she has a night-fit she generally dreams that she is falling, and on waking finds her tongue severely bitten, and feels bruised in the limbs. Emotions have no influence on the occurrence of these attacks. She sleeps very badly, and has horrible dreams, especially about the dead. She often suffers from headache and giddiness. Appetite indifferent; bowels habitually costive. She has lost a good deal of flesh lately, and presents a worn and anæmic appearance. She suffers from palpitations of the heart. The catamenia are pretty regular, although pale and scanty; a short time before the period comes on the fits are more frequent and severe.

The patient was put on a course of phosphorus, tincture of henbane, and cod-liver oil, under which the attacks steadily diminish in number and severity. The last occurred on May 7th. Her strength and general appearance were then very much improved, and she went on favorably till June 29th, when she was seized with a sharp attack of hay fever, of which she has been suffering every summer for the last seven or eight years. There was burning in the eyes, catarrh in the nose, a feeling of rawness in the throat, and very considerable dyspnœa. During the day she was obliged to stop in-doors and have the room shaded and the windows closed, but she could go out in the evening.

On July 11th, the patient being then very restless and worn out by fits of sneezing and dyspnœa, an epileptic attack occurred, in the day-time, after a free interval of sixty-four days. On the 12th she had another attack, during the early part of the night. She was very weak and low-spirited. A draught, containing five minims of the dilute hydrocyanic acid and fifteen minims of tincture of Indian hemp, was then given, in mucilage, twice a day, and the other medicines were discontinued. The distress from the hay fever was now at once greatly diminished, and after taking the draught for four days it quite ceased. No further epileptic attacks took place; and the patient got quite well again under the use of nerve-tonics.

She was seen on September 26th, being then in excellent health and spirits, and having had nothing to complain of in the interval. It remains to be seen whether the attacks have finally ceased; yet the prejudicial influence of hay fever on the progress of this, as of other similar cases, is obvious.

Congenial phimosis has been observed in eleven out of twenty-five consecutive male cases of epilepsy admitted at the infirmary. That such a frequent complication of eplepsy should hitherto have remained unnoticed can only be explained by the circumstance that epileptic patients seldom come under the eye of surgeons, and that physicians usually neglect to examine the sexual organs. The effects of congenial phimosis on the system are usually quite disregarded, although there can be little doubt that this malformation has a considerable pathological importance. There is always an accumulation of sebum between the prepuce and the gland in such cases, and herpes and balanitis may be the consequence. This irritation often leads to great sexual excitement about the period of puberty, and to masturbation, with all its consequent evil effects; frequent emissions of semen at night may also be traced to the same cause. A variety of cerebral symptoms may then be induced, such as pain in the head, giddiness, noises in the ears, eructations, sickness, &c.; which, where they depend only upon this condition, may be entirely removed by circumcision. Whether actual epileptic fits are ever the consequence of phimosis seem doubtful; yet the propriety of the operation in cases of that kind cannot be questioned, as all sources of irritation should, on principle, be removed in convulsive disorders. Several of these cases which were admitted at the infirmary have been operated upon by Mr. Solly and Mr. Spencer Wells. In no instance, however, have the fits ceased immediately, consequent upon the operation; so that a relation as between cause and effect could not have existed between phimosis and epilepsy. Yet, Dr. Althaus said, it generally seemed as if the convulsive disorder, after circumcision in such cases, yielded more readily to the remedies employed than it had done before.—*London Lancet.*

